# Shock Waves in the Treatment of Muscle Hypertonia and Dystonia

**DOI:** 10.1155/2014/637450

**Published:** 2014-09-17

**Authors:** Laura Mori, Lucio Marinelli, Elisa Pelosin, Antonio Currà, Luigi Molfetta, Giovanni Abbruzzese, Carlo Trompetto

**Affiliations:** ^1^Department of Neuroscience, Rehabilitation, Ophthalmology, Genetics, Maternal and Child Health, University of Genova, Largo Daneo 3, 16132 Genova, Italy; ^2^Department of Medical-Surgical Sciences and Biotechnologies, Sapienza University of Rome, Polo Pontino, Via Firenze, 04019 Terracina, Italy

## Abstract

Since 1997, focused shock waves therapy (FSWT) has been reported to be useful in the treatment of muscle hypertonia and dystonia. More recently, also radial shock wave therapy (RSWT) has been successfully used to treat muscle hypertonia. The studies where FSWT and RSWT have been used to treat muscle hypertonia and dystonia are reviewed in this paper. The more consistent and long lasting results were obtained in the lower limb muscles of patients affected by cerebral palsy with both FSWT and RSWT and in the distal upper limb muscles of adult stroke patients using FSWT. The most probable mechanism of action is a direct effect of shock waves on muscle fibrosis and other nonreflex components of muscle hypertonia. However, we believe that up to now the biological effects of shock waves on muscle hypertonia and dystonia cannot be clearly separated from a placebo effect.

## 1. Introduction

Muscle hypertonia is a common finding in patients with upper motor neuron syndrome (UMNS) following stroke, multiple sclerosis, spinal cord injury, and traumatic brain injury. In these patients, the increase of muscle tone has been related to the presence of spasticity, commonly defined as “a motor disorder characterised by velocity-dependent increase in the tonic stretch reflexes (“muscle tone”) with exaggerated tendon jerks resulting from hyperexcitability of the stretch reflex” [1]. Spasticity in patients with UMNS is a major cause of disability affecting daily activities and quality of life [2].

However, in addition to spasticity, muscle contracture, in an early stage after brain or spinal cord insult, and joint retraction, in a later stage, make a significant contribution to muscle hypertonia [3]. Loss of sarcomeres with muscle shortening, degenerative changes at the myotendinous junction, and accumulation of intramuscular connective and fat tissues are the basic mechanisms of muscle contracture [4]. Proliferation of fibrofatty connective tissue within the join space and adhesions of sinovial folds are involved in joint retraction. Both muscle contracture and joint retraction are secondary effects to muscle immobilisation in a shortened position [4]. Besides muscle contracture and joint retraction, another possible cause of muscle hypertonia is the impossibility of achieving a complete muscle relaxation. Such muscle overactivity, not induced by muscle stretch (spastic dystonia), has been frequently reported in patients with UMNS [4]. Therefore, muscle hypertonia in patients with UMNS can be divided into two components: hypertonia mediated by the stretch reflex (reflex muscle hypertonia), which corresponds to spasticity, and hypertonia due to muscle contracture, joint retraction, or nonreflex muscle overactivity (intrinsic or nonreflex muscle hypertonia). While it is difficult in a clinical setting to distinguish reflex and nonreflex contributions to muscle hypertonia, biomechanical measures combined with EMG recordings can provide an objective separation of these two components (for a review see Biering-Sørensen, 2006 [5]).

Dystonia is a syndrome dominated by sustained muscle contractions, frequently causing twisting and repetitive movements or abnormal postures [6]. Dystonia can be primary (or idiopathic) and secondary (or symptomatic). Most lesions responsible for secondary dystonia involve the caudate nucleus, the putamen, the globus pallidus, and the thalamus. As the lesions in these sites share the capability of altering the thalamic signalling to the frontal cortex, dystonia is thought to originate from a dysfunction of the motor cortex induced by the derangement of thalamocortical connections [7–9].

Since the late 1980s, focused shock waves therapy (FSWT) has been widely and successfully used in the treatment of pain in various musculoskeletal disorders [10]. FSWT devices use pressure waves generated through electromagnetic, electrohydraulic, and piezoelectric sources. These waves have their point of higher pressure in the focus, which is placed within the treated tissue [11, 12]; for this reason they are defined as focused shock waves.

In 1999, a new technology using a ballistic source to generate pressure waves was introduced. This technology is called radial shock wave therapy (RSWT). The ballistic source consists of a tube within which compressed air (1–4 bar) is used to fire a bullet that strikes a metal applicator placed on the patient's skin. The applicator transforms the kinetic energy of the bullet into radially expanding pressure waves with a low penetration power (less than 3 cm). These unfocused shock waves have their point of highest pressure at the tip of the applicator, outside the treated tissue [11, 12].

It has been shown that both focused (FSWT) and unfocused (RSWT) shock waves produce cavitation bubbles in the treated tissue [12]. The cavitation is consequent to the negative phase of the wave propagation. The rapid collapse of the cavitation bubbles leads to secondary pressure waves. Cavitation-mediated mechanisms could have a central role in the action of both FSWT and RSWT for the musculoskeletal system [12].

From a theoretical point of view, shock waves could be useful to treat dystonia and muscle hypertonia in patients with UMNS. In accordance with the effects on tendon diseases [10], shock waves could have a direct effect on muscle fibrosis and other nonreflex components of muscle hypertonia, which are likely to be present also in some dystonic patients [8]. Furthermore, shock waves acting at the muscular level could modify the sensory inflow from the treated muscle to the spinal cord, thus reducing spinal cord excitability and mitigating spasticity.

The objective of the present review is to provide an overview on the treatment of dystonia and muscle hypertonia using shock waves. For the preparation of this paper, a literature survey in the PubMed electronic database was performed and covered the period from January 1985 to April 2014. We selected all the studies in humans dealing with shock wave therapy and muscle hypertonia/dystonia without restrictions. We found eight studies in which FSWT has been used to treat muscle hypertonia [13–20] and one study using FSWT to treat dystonia [21]. In three studies, RSWT has been used to treat muscle hypertonia [22–24]. In one study, aimed at investigating the action mechanisms of shock waves in patients with muscle hypertonia, FSWT has been delivered to healthy subjects to evaluate the effect of shock waves on the peripheral and central nerve conduction [25] (Table 1).

## 2. FSWT to Treat Hypertonia in Patients with Cerebral Palsy (CP)

Lohse-Busch et al. were the first to use shock waves in an open-label single-arm study to treat muscle hypertonia in children and young adults with CP (33 patients), tetraparesis after trauma (1 patient), kernicterus with athetosis (1 patient), and arthrogryposis multiplex congenita (1 patient) [14]. Shock waves were delivered to lower limb muscles affected by contractures (hamstrings, triceps surae, and ileopsoas). To disperse the shock waves throughout the muscles, their focus (intensity = 0.06 mJ/mm^2^) was placed either within the coupling cushion of the therapy source (Minilith SL1, Storz Medical AG) or obliquely outside the patient's body. Shock waves were delivered in a single session and each muscle was treated with 500 shots. The patients received also physiotherapy. The outcome measure was the passive range of movement (ROM) of hip, knee, and ankle joints. The main result was that passive ROM increased after the treatment for several weeks (at least 3-4 weeks). Positive clinical effects were also found in muscle hypertonia, cocontractions, dyskinetic, and ataxic symptoms.

More recently, Amelio and Manganotti investigated, in a placebo-controlled study, the effect of a single session of FSWT in 12 children with CP showing a unilateral spastic equinovarus foot [13]. The shock waves (Modulith SLK, Storz Medical AG) were delivered to the hypertonic triceps surae (1500 shots to each of the 3 muscles, 4500 shots in total) using an intensity of 0.03 mJ/mm^2^. Each patient was treated with placebo followed 6 weeks later by FSWT. The placebo treatment was applied with the identical instrumentations used for FSWT and with the same sound, but without shock wave energy. The patients were evaluated immediately before and after the treatment (both placebo and active). The patients were also evaluated 1, 4, and 12 weeks after FSWT. No physiotherapy was performed after FSWT. The outcome measures were passive ROM of ankle joint, the Modified Ashworth Scale (MAS) for ankle plantar flexors, and a baropodometric assessment. After FSWT, muscle tone was reduced and passive ROM was increased. The baropodometric evaluation revealed an increase in the whole plantar surface area of the treated limb. These effects persisted 4 weeks after FSWT, but they were not present 12 weeks after. No changes were observed after placebo treatment. No side effects were observed in any patient [13].

## 3. RSWT to Treat Hypertonia in Patients with Cerebral Palsy (CP)

In 2011, Vidal et al. assessed the efficacy of 3 sessions of RSWT (1 session per week; intensity: 2 Bars; 2000 shots for each muscle) in 15 CP patients [24]. RSWT was delivered by means of a Swiss Dolorclast (EMS-Switzerland) device. The study focused on 40 spastic muscles (forearm and wrist flexors; hip adductors; soleus and hamstrings), which were divided into 3 groups. In group I (14 muscles), RSWT was delivered only to the spastic muscles; in group II (13 muscles), RSWT was delivered to the spastic muscles and their antagonists; in group III (13 muscles), placebo stimulation was delivered only to the spastic muscles. The characteristics of placebo stimulation were not specified. In the methods, it was only reported that patients received placebo “via application of a sham RSWT with sound.” The outcome measure for upper limb muscles was hypertonia measured with the Ashworth scale (AS), while for lower limb muscles the outcome measure was the passive ROM. Patients were evaluated just before the treatment and 1, 2, and 3 months after the last session. One month after RSWT, AS scores decreased in group I muscles and passive ROM values increased in both group I and group II muscles. No differences were found between group I and group II muscles and no changes were found after placebo stimulation (group III). The results were maintained 2 months after the last session of RSWT, while 3 months after they were no longer present. No major side effects were reported [24].

Gonkova et al. [22] used a single session of RSWT to treat the flexor muscles of the ankle in 25 children with CP (intensity: 1.5 Bars; 1500 shots for each of the 3 muscles forming the triceps surae). Forty ankle flexor muscle groups were treated using a BTL-5000 unit (BTL Columbia, South Carolina, USA). The outcome measures were muscle hypertonia, measured with the MAS, passive ROM, and a baropodometric assessment. MAS scores and ROM values were collected before RSWT, immediately after RSWT, and 2 weeks and 4 weeks after RSWT. The baropodometric assessment was performed before and immediately after RSWT. Each subject was treated first with placebo stimulation and, a month later, with the shock waves. For the placebo treatment, two cushions were inserted between the applicator and the patient's skin and 100 shots were applied with the lowest intensity. After RSWT, no other physiotherapy was performed in the following 4 weeks. The results showed that the reduction of MAS scores and the increase of ROM values were present just after RSWT and lasted 4 weeks. The baropodometric assessment revealed an increased peak pressure under the heel and an increase of the foot contact area after RSWT. No effects were found after the placebo stimulation. The authors said that the RSWT was “well-tolerated”; no other comments on possible side effects were reported in the paper [22].

## 4. FSWT to Treat Hypertonia in Patients with Stroke: Upper Limb Studies

In 2005, Manganotti and Amelio reported the effect of a single session of FSWT in 20 adult patients with poststroke spasticity. This was a placebo-controlled study, in which each patient was first treated with placebo and then, after 1 week, the shock waves were applied. Shock waves were delivered to flexor muscles in the forearm (flexor ulnaris and flexor radialis, total 1500 shots) and intrinsic muscles of the hand (interosseus muscles, total 3200 shots) using an energy of 0.03 mJ/mm^2^ (Modulith SLK, Storz Medical AG). Different points of application were used to treat several areas of the hypertonic muscles. Placebo treatment was applied with the same instrumentations and the same sound, but without shock wave energy. The outcome measures were muscle tone of wrist and fingers flexors, evaluated using the AS and passive ROM. Motor nerve conduction velocity and F-waves from abductor digiti minimi (ADM) were also recorded. Patients were evaluated before placebo, immediately after placebo, before FSWT (1 week after placebo), and 1, 4, and 12 weeks after FSWT. In addition, a needle electromyography (EMG) recording in the first interosseus muscle was performed 4 weeks after the treatment. Muscle tone was reduced in wrist flexors 1 week and 4 weeks after FSWT, while in fingers flexors muscle tone was reduced 1, 4, and 12 weeks after shock waves. Passive ROM was increased 1 week and 4 weeks after FSWT in both wrist and fingers flexors. Muscle tone and passive ROM did not change after placebo. No changes were found in the electrophysiological parameters both after FSWT and placebo. No adverse effects were observed in any patient [15].

In 2013, Troncati et al., in an open study without a placebo arm, used two sessions of FSWT (1 week between the first and the second session) to treat the hypertonic flexor muscles in the forearm (1600 shots for each treated muscle) and interosseus muscles of the hand (800 shots for each muscle) of 12 adult stroke patients. Shock waves were applied to flexor muscles using an energy of 0.105 mJ/mm^2^, while for intrinsic hand muscles an energy of 0.08 mJ/mm^2^ was used (Modulith SLK, Storz Medical AG). Patients were examined at baseline, immediately after FSWT, and 1 week, 3 months, and 6 months after FSWT. Muscle tone of upper limb muscles was assessed with MAS; furthermore, the subscores of the upper limb section of the Fugl-Meyer Scale (FM) for motricity, passive ROM, and pain were used. Immediately after the treatment, muscle tone was reduced (not only in the treated muscles, but also in the shoulder external rotators and in the flexors of the elbow) and FM subscores improved. The effects on MAS, passive ROM, and motricity subscores persisted for 3 and 6 months after FSWT. The possible adverse effects have not been discussed in the paper [20].

The SBOTE (Spasticity treated by Botulinum Toxin and ESWT) study was designed to compare, in 32 stroke patients, the effectiveness of the following 2 treatments for upper limb spasticity: Botulinum Toxin A (BoNT-A) + FSWT versus BoNT-A + Electrical Stimulation (ES) [18]. BoNT-A was injected in the flexor digitorum superficialis (FDS) muscle. In 16 patients, immediately after BoNT-A injection, ES (5 Hz of rectangular biphasic balanced current at the intensity of 50–90 mA) was applied on the FDS belly for 30 minutes twice a day for 5 days. In the other 16 patients, immediately after BoNT-A injection, FSWT was delivered once a day for 5 days. Shock waves were focused on the forearm (2000 shots on the FDS belly and its proximal muscle-tendon junction) using an energy of 0.03 mJ/mm^2^ (Modulith SLK, Storz Medical AG). The outcome measures were MAS and VAS for pain and frequency of muscle spasms. Patients were evaluated before and after the treatment (15, 30, and 90 days after). In the patients treated with FSWT, muscle tone reduction persisted 30 days after the treatment and the reduction of the frequency of muscle spasms persisted 90 days after the treatment. The reduction of pain was significant 30 days after the treatment. The results obtained in the patients treated with ES were significantly shorter in duration and smaller in size. None of the patients reported adverse effects [18].

## 5. FSWT to Treat Hypertonia in Patients with Stroke: Lower Limb Studies

The study of Sohn et al. [19] was designed to investigate the mechanisms of FSWT on muscle hypertonia. The clinical effects induced by shock waves were evaluated only immediately after the treatment. This study will be described in Section 8.

In 2013, Moon et al. used FSWT to treat the hypertonic ankle plantar flexor muscles in 30 stroke patients. Three sessions of FSWT, generated by a piezoelectric source (Richard Wolf GmbH), were delivered to the medial and lateral gastrocnemius (1500 shots) using an energy of 0.089 mJ/mm^2^. A single session of placebo treatment, provided using the same equipment used for FSWT avoiding contact between the device and the subject's skin, was performed 1 week before FSWT. Physiotherapy was performed during the study. The clinical outcome measures include the MAS and the passive ROM. Furthermore, a biomechanical assessment of muscle hypertonia was made using an isokinetic dynamometer. The peak value of the eccentric torque occurring during passive joint movements (Peak Eccentric Torque, PET) and the joint angle at which the eccentric torque begins to occur (Torque Threshold Angle, TTA) were measured. No changes were found after placebo treatment. MAS scores and PET and TTA values decreased immediately after FSWT. MAS scores and PET values also decreased 1 week after FSWT, but such results were no longer present 4 weeks after FSWT [16].

Very recently, Santamato et al. applied a single session of FSWT to treat the hypertonic ankle plantar flexor muscles in 23 adult stroke patients using an open uncontrolled study design [17]. Shock waves at the energy of 0.10 mJ/mm^2^ were applied using EvoTron RFL0300 (Sanuwave AG). A number of 1500 shots were used to treat each gastrocnemius muscle and soleus muscle. Each patient was evaluated at baseline, immediately after FSWT, and 30 days after FSWT. No physiotherapy was performed after FSWT. The clinical outcome measures were the MAS and the passive ROM. At baseline, the patients underwent a muscle ultrasound evaluation of ankle plantar flexors and muscle echogenicity was measured using the Heckmatt scale. Furthermore, at baseline and 30 days after FSWT, distal motor nerve conduction velocity and F-waves responses from abductor hallucis elicited by tibial nerve stimulation were recorded. Immediately after the treatment, there was a decrease of MAS score and an increase of passive ROM. These results persisted 30 days after only in those patients with baseline echogenicity level of I, II, and III on the Heckmatt scale. After FSWT, the neurophysiological assessment did not show any significant change in nerve conduction velocity and spinal excitability. Mild adverse effects lasting only a few days were reported (pain in 5 subjects and local weakness in 2 subjects). This study suggests that FSWT does not produce long-lasting effects when the muscle is atrophic and replaced by fat and fibrotic tissue [17].

## 6. RSWT to Treat Hypertonia in Stroke Patients: A Study in the Upper Limb

In 2013, Kim et al. used 5 sessions of RSWT (1 session every 2 or 3 days; intensity: 1.6 Bar; 3000 pulses per session) to treat the spastic subscapularis muscles in 57 stroke patients. The device used was Masterplus MP 200 (Storz Medical AG, Tagerwillen, Switzerland). The outcome measures were passive ROM and the MAS (shoulder external rotation). Furthermore, shoulder pain was evaluated in a subgroup of 18 patients with a minimental status examination (MMSE) > 24 and a visual analogue scale (VAS) for shoulder pain > 4/10. Patients were evaluated at the start of each session (5 times) and once a week after the last session for a period of six weeks (6 times). During the period of the study, patients received physiotherapy including massage, passive muscle stretching, and active exercises. The study was not controlled versus placebo. During the 5 sessions of RSWT and in the subsequent 4 weeks, MAS and VAS scores for shoulder pain were lower and passive ROM values were higher, compared to baseline values. Four weeks after the last session the effects decreased. No serious side effects were observed over the 6 weeks follow-up period [23].

## 7. FSWT to Treat Dystonia

Trompetto et al. used FSWT to treat dystonia in a small group of subjects: 3 patients with secondary dystonia due to a lesion in the basal ganglia and 3 patients with writer's cramp [21]. Shock waves were delivered in 4 sessions (once weekly), using an average intensity of 0.03 mJ/mm^2^ (Modulith SLK, Storz Medical AG). In patients with secondary dystonia, the muscles responsible for the dystonic movements were identified and treated (800 shots for the selected intrinsic hand muscles and 2000 shots for the selected flexor and extensor muscles in the forearm). In the 3 subjects with writer's cramp, FSWT was widely distributed to the volar (3000 shots) and dorsal (3000 shots) surfaces of the forearm. Each patient was treated with a single session of placebo 2 weeks before FSWT. In the subjects with secondary dystonia, the outcome measures were the unified dystonia rating scale (UDRS) and a four-point pain intensity scale. In the subjects with writer's cramp, the arm dystonia disability scale (ADDS) was used. In addition, before the first and after the last session of FSWT, somatosensory evoked potentials obtained by median nerve stimulation and motor nerve conduction velocity with F-waves from ADM muscle were recorded in each patient. While placebo treatment left unchanged the clinical scores in all the subjects, after FSWT the 3 patients with secondary dystonia showed a marked improvement of both dystonia and pain lasting at least 1 month after the last session. In the 3 patients with writer's cramp, the improvement after FSWT was less consistent being effective only in two subjects. No significant changes of electrophysiological values were found. Shock waves induced side effects in none of the subjects, including weakness in the treated muscles [21].

## 8. Neurophysiological Studies

In 2011, Sohn et al. used a single session of FSWT (Evotron, SwiTech Medial AG), delivered to the medial gastrocnemius muscle (1500 shots), in 10 adult poststroke hemiplegic patients with hypertonia in ankle plantar flexor muscles (triceps surae) using an energy of 0.1 mJ/mm^2^. The subjects were investigated before and immediately after the treatment. The outcome clinical measures were the MAS score of ankle plantar flexors muscles. To study spinal excitability, F-waves in the abductor hallucis muscle and H-reflexes in the soleus muscle were recorded. After the treatment, the MAS scores were reduced. Mild pain was produced by the treatment. The absence of a significant effect on spinal excitability was discussed since F-wave minimal latency, H-reflex latency, and H-reflex/M-wave ratio remained stable before and after the treatment [19].

In a group of ten healthy subjects, Manganotti et al. [25] investigated the effects on peripheral nerve conduction and corticospinal excitability induced by a single session of FSWT applied to the right ADM muscle (1600 shots at energy of 0.03 mJ/mm^2^, delivered by Modulith SLK-Storz Medical AG). Motor nerve conduction velocity and F-waves were recorded from right ADM muscle by ulnar nerve stimulation, sensory action potentials were recorded from the right V finger by ulnar nerve stimulation, and motor potentials were evoked by transcranial magnetic stimulation in the right ADM muscle. The subjects were investigated before and after FSWT (immediately, 15 and 30 minutes after FSWT). No changes were found in any of the investigated parameters [25].

## 9. Discussion

### 9.1. Shock Waves Effectiveness and Safety

The 4 studies in CP patients (2 studies with FSWT and 2 studies with RSWT) provided rather consistent results. In all the studies, the treatment was applied to the lower limb muscles. Only in one study [24] the shock waves were also applied to the upper limb muscles. Only one study was a multiple sessions study [24], while the others were single session studies. All the studies but one [14] were placebo controlled. In all the 4 studies muscle hypertonia was evaluated clinically and muscle hypertonia was reduced for at least 3-4 weeks after the treatment. In the 2 studies where a complete time course of the shock waves effects was assessed, 3 months after the treatment the benefit was no longer statistically significant [13, 24]. Taken together, these studies demonstrate that the reduction of muscle tone after shock waves in CP patients lasted more than 3-4 weeks and less than 3 months.

Similarly, the 3 studies in which FSWT was used to treat hypertonic upper limb muscles in adult stroke patients gave consistent results. Only one of them [15] was placebo-controlled. Two were multiple sessions studies [18, 20], while the remaining one was a single session study. In all the 3 studies, the shock waves were focused on the flexor muscles in the forearm and in the intrinsic hand muscles. The effects were long lasting since the reduction of muscle hypertonia or muscle spasms, evaluated clinically, was still present 3 months [15, 18] and even 6 months [20] after the treatment.

The results obtained in the 2 studies in which FSWT was applied to the lower limb muscles of adult stroke patients were less relevant. The multiple sessions, placebo-controlled study of Moon et al. [16] was the only study treating hypertonia with shock waves in which muscle tone was measured not only through clinical, but also through a biomechanical assessment. The reduction of muscle hypertonia lasted only 1 week after the treatment; 4 weeks after FSWT the effect was not significant. In the other study, a single session of FSWT produced a reduction of muscle hypertonia 4 weeks after the treatment only in those patients without severe muscle secondary changes [17].

In the study performed using RSWT in the adult stroke patients, the treatment was focused on the subscapularis muscle. This was a multiple sessions study without placebo. The clinical evaluation revealed an improvement of muscle tone, which was fairly stable during the 4 weeks after the last session; however the effect decreased during the following weeks.

A study performed in a small sample of subjects showed that FSWT can be useful in patients with secondary dystonia, reporting a reduction of involuntary muscle contractions and pain in all the tested subjects for at least 1 month. Inconsistent results were obtained in patients with writer's cramp [21].

No major adverse effects were observed in any study. Only mild and short-lasting adverse effects were reported, including pain, local weakness, small superficial hematomas, and petechiae [17, 24].

### 9.2. The Mechanisms of Action

Theoretically, shock waves could reduce the excitability of the stretch reflex altering the nerve conduction of sensory and motor fibers connecting the treated muscle to the spinal cord. Furthermore, the mechanical stimulation by shock waves on the muscle fibers adjacent to the tendon could decrease spinal excitability [15, 17]. However, the neurophysiological assessments performed before and after FSWT in patients with muscle hypertonia [15, 17, 19], in patients with dystonia [21], and in healthy volunteers [25] did not find any changes in motor and sensory peripheral nerve conduction and in corticospinal excitability. The lack of changes in peripheral conduction and spinal excitability suggests that the most probable mechanism of action is a direct effect of shock waves on muscle fibrosis and other nonreflex components of muscle hypertonia. Through its action on nonreflex hypertonia, however, shock waves could also reduce spasticity. The reduced extensibility due to soft tissue changes causes any pulling force to be transmitted more readily to the muscle spindles [26]. In this condition, an exaggerated spindle discharge in response to muscle stretch results in an increased stretch reflex [4]. Thus, the reduction of nonreflex hypertonia could modify muscle spindles excitability, leading to a secondary reduction of spasticity.

### 9.3. The Placebo Effect

The major limitation of the reviewed studies is represented by placebo treatment. Six studies were not placebo controlled [14, 17–20, 23]. In the remaining 6 studies, the placebo treatments were applied using the same instrumentations used for FSWT or RSWT, but without shock wave energy [13, 15, 24] or using a method to prevent the shock waves from reaching the target muscles [16, 21, 22]. Differently from pharmacological studies, the critical issue concerning physical treatments such as shock waves is the difficulty in making the placebo treatment actually effective. How credible can a shock wave treatment be without shock waves? Reasonably it could be argued that the absence of clinical effects after the placebo treatment may be the consequence that some patients guessed that they were not subjected to an effective treatment. This obviously limits the value of the results obtained in the placebo arm and does not allow estimating the amount of placebo effect in the real treatment arm. Furthermore, in all the placebo controlled studies but one [24] the same patients received the placebo treatment in the first place and then the real treatment. In such way, patients could not only have suspected to have been treated with placebo during the first treatment, but also have probably realized that they had been treated with shock waves during the second treatment.

## 10. Conclusions

Shock waves seem to be useful in treating muscle hypertonia in UMNS patients. The more consistent and encouraging results were obtained in CP patients with both FSWT and RSWT and in the distal upper limb muscles of adult stroke patients using FSWT. However, we believe that up to now the biological effects of shock waves on muscle hypertonia cannot be clearly separated from a placebo effect. New solutions are needed to address this issue.

## Figures and Tables

**Table 1:** 

Reference	Participants	Treatment	Treated muscles	Outcome measures	Placebo	Other treatments	Results	Side effects
Lohse-Busch et al. 1997 [14]	35 children and young adults with CP	FSWT: 1 session; 0.06 mJ/mm^2^; 500 shots for each treated muscle	Hamstrings, Triceps surae and Ileopsoas	ROM	No	Physiotherapy	↑ ROM lasting ≥3 weeks	No

Amelio and Manganotti, 2010 [13]	12 children with CP	FSWT: 1 session; 0.03 mJ/mm^2^; 4500 shots in total	Triceps surae	ROM, MAS, baropodometry	1 session of placebo followed 6 weeks later by FSWT	No	↑ ROM, ↓ MAS scores, and ↑ plantar surface area lasting ≥4 weeks and <12 weeks after FSWT. No effects after placebo	No

Vidal et al. 2011 [24]	15 children and adults with CP	RSWT: 3 sessions (1/week); 2 Bars; in each session, 2000 shots for each treated muscle	Forearm and wrist flexors; hip adductors; soleus and hamstrings	AS for upper limb muscles; ROM for lower limb muscles	Placebo was applied to 13 muscles (patients' number not specified)	Not specified	↓ AS scores and ↑ ROM lasting ≥8 weeks and <12 weeks after FSWT. No effects after placebo	Petechiae and light pain during RSWT in 3 patients

Gonkova et al. 2013 [22]	25 children with CP	RSWT: 1 session; 1.5 Bars; 4500 shots in total	Triceps surae	ROM, MAS, and baropodometry	1 placebo session, then RSWT after 1 month	No	↓ MAS scores and ↑ ROM lasting ≥4 weeks after RSWT. No effects after placebo	No

Manganotti and Amelio, 2005 [15]	20 adults with poststroke spasticity	FSWT: 1 session; 0.03 mJ/mm^2^; 4700 shots in total	Wrist flexors; interosseus muscles of the hand	ROM, AS, MCV, and F-waves from ADM; EMG (FDI)	1 placebo session followed by FSWT after 1 week	Not specified	↑ ROM lasting 4–12 weeks after FSWT. ↓ AS scores lasting 4–12 weeks in wrist flexors and lasting ≥12 weeks in fingers flexors after FSWT. No changes of MCV, F-waves, and EMG after FSWT. No effects after placebo	No

Troncati et al. 2013 [20]	12 adults with poststroke spasticity	FSWT: 2 sessions (1 session per week); 0.08/0.105 mJ/mm^2^; 800/1600 shots for each treated muscle	Flexor muscles in the forearm; interosseus muscles of the hand	MAS, subscores of the upper limb FM scale for motricity, ROM and pain; perceived benefit (VAS)	No	Not specified	↑ ROM and motricity subscores and ↓ MAS scores lasting ≥6 months. ↓ Pain lasting 1–12 weeks. No changes in perceived benefit (VAS)	No

Santamato et al. 2013 [18]	16 adults with poststroke spasticity	FSWT: 5 sessions (1 session every day starting from BoNT-A injection); 0.03 mJ/mm^2^; 2000 shots in total	Flexor digitorum superficialis muscle	MAS; VAS for pain and frequency of muscle spasms	No	BoNT-A	↓ MAS scores and ↓ pain lasting 1–3 months. ↓ Frequency of muscle spasms lasting ≥3 months. In 16 patients treated with BoNT-A and electrical stimulation, the results were shorter in duration and smaller in size	No

Moon et al. 2013 [16]	30 adults with poststroke spasticity	FSWT: 3 sessions (1 session per week); 0.089 mJ/mm^2^; 1500 shots in total	Medial and lateral gastrocnemius	ROM, clonus score, FM assessment of the lower limb, MAS, biomechanical assessment (PET and TTA)	1 placebo session followed by FSWT	Physiotherapy	↓ MAS scores and PET values lasting 1–4 weeks. No effects after placebo	No

Santamato et al. 2014 [17]	23 adults with poststroke spasticity	FSWT: 1 session; 0.10 mJ/mm^2^; 4500 shots in total	Triceps surae	ROM, MAS, MCV, and F-waves from AH	No	No	↓ MAS scores and ↑ ROM lasting ≥1 month after FSWT in the patients with baseline HS <4. No long-lasting effects in patients with HS = 4. No MCV and F-waves changes after FSWT	Pain in 5 subjects and local weakness in 2 subjects

Kim et al. 2013 [23]	57 adults with poststroke spasticity	RSWT: 5 sessions (1 session every 2 or 3 days); 1.6 Bars; in each session, 3000 shots in total	Subscapularis muscle	ROM, MAS, VAS for pain	No	Physiotherapy	↓ MAS scores, ↓ pain, and ↑ ROM lasting 4 weeks	Petechiae in 1 patient and light pain during RSWT in some patients

Trompetto et al. 2009 [21]	3 adults with secondary dystonia and 3 with writer's cramp	FSWT: 4 sessions (1 session per week); 0.03 mJ/mm^2^; up to 8000 shots	Forearm and hand muscles	UDRS, ADDS, 4-point pain intensity scale, median nerve SEPs, F-waves from ADM	1 placebo session followed by FSWT after 2 weeks	No	In patients with secondary dystonia, clinical benefit on dystonia and pain lasting 1-2 months. Less consistent effects on writer's cramp patients. No changes of SEPs and F-waves after FSWT. No effects after placebo	No

Sohn et al. 2011 [19]	10 adults with poststroke spasticity	FSWT: 1 session; 0.10 mJ/mm^2^; 1500 shots in total	Medial gastrocnemius muscle	MAS, F-waves from AH and H-reflex from soleus	No	No	↓ MAS scores just after FSWT (no follow-up). No changes of F-waves and H-reflex	Mild pain during FSWT

Manganotti, et al. 2012 [25]	10 healthy subjects	FSWT: 1 session; 0.03 mJ/mm^2^; 1600 shots in total	Right ADM	MCV, F-waves, and MEPs from right ADM; SAP from 5° finger by ulnar nerve stimulation	No	No	No changes in any of the investigated parameters	No

ADDS: arm dystonia disability scale; ADM: abductor digiti minimi; AH: abductor hallucis; AS: Ashworth scale; BoNT-A: botulinum toxin type A; CP: cerebral palsy; EMG: electromyography; FDI: first dorsal interosseus; FM: Fugl-Meyer; FSWT: focused shock waves therapy; HS: Heckmatt scale; MAS: modified Ashworth scale; MCV: motor conduction velocity; MEPs: motor evoked potentials; PET: peak eccentric torque; ROM: passive range of movement; RSWT: radial shock waves therapy; SAP: sensory afferent potential; SEPs: sensory evoked potentials; TTA: torque threshold angle; UDRS: unified dystonia rating scale; VAS: visual analogue scale.
